# Endoplasmic reticulum and lysosomal Ca^2+^ stores are remodelled in *GBA1*-linked Parkinson disease patient fibroblasts

**DOI:** 10.1016/j.ceca.2015.11.002

**Published:** 2016-01

**Authors:** Bethan S. Kilpatrick, Joana Magalhaes, Michelle S. Beavan, Alisdair McNeill, Matthew E. Gegg, Michael W.J. Cleeter, Duncan Bloor-Young, Grant C. Churchill, Michael R. Duchen, Anthony H. Schapira, Sandip Patel

**Affiliations:** a Department of Cell and Developmental Biology, University College London, Gower Street, London WC1E 6BT, UK; b Department of Clinical Neurosciences, Institute of Neurology, University College London, London NW3 2PF, UK; c Department of Pharmacology, University of Oxford, Oxford OX1 3QT, UK

**Keywords:** ER, endoplasmic reticulum, cADPR, cyclic ADP-ribose, SERCA, sarco-endoplasmic reticulum Ca^2+^ ATPase, PD, Parkinson disease, GD, Gaucher disease, ASX, asymptomatic, CBE, conduritol B epoxide, GPN, glycyl-l-phenylalanine 2-naphthylamide, LAMP, lysosome associated membrane protein, LC3, microtubule-associated protein 1A/1B-light chain 3, Ca^2+^, Endoplasmic reticulum, Lysosomes, Parkinson disease, Gaucher disease

## Abstract

Mutations in β-glucocerebrosidase (encoded by *GBA1*) cause Gaucher disease (GD), a lysosomal storage disorder, and increase the risk of developing Parkinson disease (PD). The pathogenetic relationship between the two disorders is unclear. Here, we characterised Ca^2+^ release in fibroblasts from type I GD and PD patients together with age-matched, asymptomatic carriers, all with the common N370S mutation in β-glucocerebrosidase. We show that endoplasmic reticulum (ER) Ca^2+^ release was potentiated in GD and PD patient fibroblasts but not in cells from asymptomatic carriers. ER Ca^2+^ signalling was also potentiated in fibroblasts from aged healthy subjects relative to younger individuals but not further increased in aged PD patient cells. Chemical or molecular inhibition of β-glucocerebrosidase in fibroblasts and a neuronal cell line did not affect ER Ca^2+^ signalling suggesting defects are independent of enzymatic activity loss. Conversely, lysosomal Ca^2+^ store content was reduced in PD fibroblasts and associated with age-dependent alterations in lysosomal morphology. Accelerated remodelling of Ca^2+^ stores by pathogenic *GBA1* mutations may therefore feature in PD.

## Highlights

• ER Ca^2+^ signalling is potentiated in PD patient fibroblasts.
• Lysosomal Ca^2+^ signalling is inhibited in PD patient fibroblasts.
• Remodelling of Ca^2+^ stores is age-dependent.

## Graphical abstract

**Figure:**
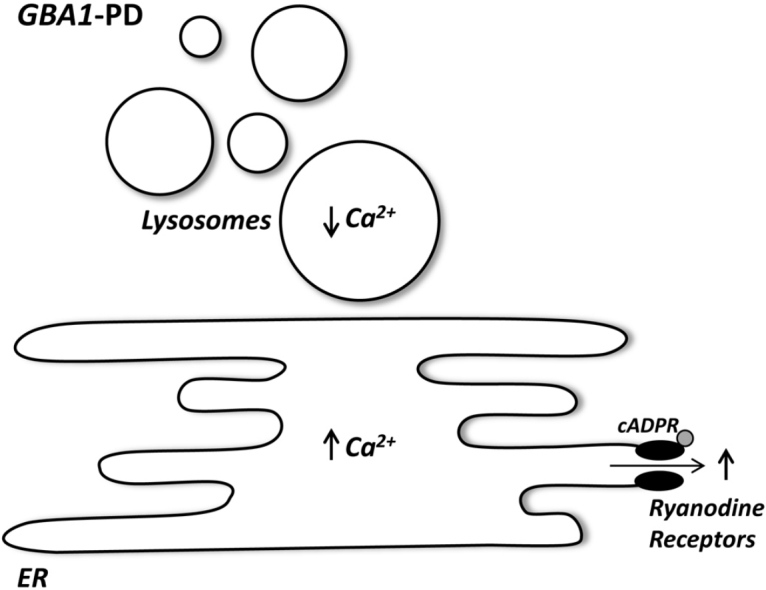

## 1 Introduction

Changes in the concentration of cytosolic Ca^2+^ form the basis of a ubiquitous signalling pathway [1]. Ca^2+^ signals derive not only from the extracellular space, but also from Ca^2+^ stores, within the cell, through the opening of intracellular Ca^2+^-permeable channels [2]. The best characterised Ca^2+^ store is the ER which houses IP_3_- and ryanodine-sensitive Ca^2+^ channels. The latter are activated by the second messenger cyclic ADP-ribose [3]. Ca^2+^ pumps (such as SERCA), exchangers and buffers act to temper Ca^2+^ increases in a highly regulated Ca^2+^ network [2]. It is becoming increasingly clear that lysosomes and other acidic organelles such as lysosome-related organelles, endosomes, secretory granules and the Golgi complex are also integral sources of Ca^2+^ [4], [5]. Lysosomes are thought to drive global Ca^2+^ signals by providing a “trigger” release of Ca^2+^ which is then amplified by Ca^2+^ channels on the ER, possibly through recently described membrane-contact sites between the two organelles [6]. ER and lysosomal Ca^2+^ stores are thus functionally and physically coupled similar to coupling between the ER Ca^2+^ stores and mitochondria/plasma membrane [7].

Gaucher disease (GD) is the most common of the lysosomal storage disorders [8]. It results due to recessive mutations in *GBA1* which encodes the lysosomal enzyme β-glucocerebrosidase responsible for hydrolysis of glucocerebroside to glucose and ceramide. Type I GD (often associated with the N370S mutation) is traditionally considered non-neuronopathic whereas types II and III are associated with neurodegeneration. But both type I GD sufferers and carriers of *GBA1* mutations are up to 20 times more likely to develop Parkinson disease (PD). Mutations in *GBA1* are therefore one of the highest known risk factors for this neurodegenerative disorder [9]. Genetic associations between PD and GD add to a body of literature implicating lysosomal dysfunction in the pathogenesis of PD [10], [11], which likely occurs upstream of established mitochondrial dysfunction [12]. The mechanism by which *GBA1* mutations mediate PD pathogenesis remains undefined. It may involve the unfolded protein response and ER stress as a consequence of mutant protein trapping or interactions with α-synuclein metabolism leading to Lewy body formation [13]. However, not all *GBA1* carriers develop PD suggesting additional pathogenic mechanisms are involved.

De-regulated Ca^2+^ signalling is established in a number of pathologies and has been implicated in both GD and PD as well as ageing, a major risk factor for neurodegenerative disease [7], [14]. ER Ca^2+^ stores appear to be hypersensitive to ryanodine receptor activation in a pharmacological neuronal model of GD resulting in sensitisation to cell death [15]. Whether lysosomal Ca^2+^ stores are affected in the disease is not known, although lysosomal Ca^2+^ content is reduced in Niemann–Pick type C1 disease [16], a distinct lysosomal storage disorder also potentially linked to PD [17]. In PD, attention has focussed mainly on Ca^2+^ influx since the affected dopaminergic neurons of the substantia nigra pars compacta exhibit unusual pace-making activity associated with influx of Ca^2+^ through L-type voltage-sensitive Ca^2+^ channels [18]. The resulting oscillations in cytosolic Ca^2+^ are thought to impose metabolic stress on the mitochondria [19], [20]. The role of ER and lysosomal Ca^2+^ stores in PD is largely unexplored.

In the present study, we identify age-dependent reciprocal changes in ER and lysosomal Ca^2+^ homeostasis in patient fibroblasts from GD and *GBA1*-linked PD sufferers. These data point to altered Ca^2+^ signalling in *GBA1*-disease and in ageing as possible contributors to PD pathology.

## 2 Methods

### 2.1 Patient fibroblasts

Primary fibroblast cultures were generated from skin biopsies as described in [21]. GD (type I) and PD patients carried the mutant allele encoding the N370S variant in β-glucocerebrosidase. The GD patient was a compound heterozygote with an additional 1263del55 mutation. For simplicity, these genotypes are referred to as *GBA1*^**mut/mut GD**^ and *GBA1*^**wt/mut PD**^, respectively. Cultures were also established from asymptomatic (ASX) non-manifesting N370S *GBA1* carriers (*GBA1*^**wt/mut ASX**^). Thus, all lines had the same mutant allele to facilitate comparison. For control purposes, fibroblasts were acquired from age-matched, apparently healthy individuals (*GBA1*^**wt/wt**^). The fibroblasts were categorised according to age. The “young” cohort were obtained from individuals under the age of 60 whereas the “aged” cohort were derived from individuals over 70 years old (exact age denoted in subscripts). A summary of fibroblasts used in this study is provided in Table S1.

### 2.2 Cell culture

Fibroblasts were maintained in DMEM. SH-SY5Y cells were maintained in a 1:1 mixture of DMEM:Ham's F12 media and 1% (v/v) non-essential amino acids (all from Invitrogen). SH-SY5Y cells with stable knock down of β-glucocerebrosidase were described in [22]. Media were supplemented with 10% (v/v) heat inactivated FBS, 100 units/ml penicillin and 100 μg/ml streptomycin. Cells were cultured at 37 °C in a humidified atmosphere with 5% CO_2_. In some experiments, cells were cultured for 7–11 days with the irreversible β-glucocerebrosidase inhibitor, conduritol B epoxide (CBE, 10 μM; Sigma–Aldrich). Media, containing CBE, was replenished every 5 (fibroblasts) or 2–3 (SH-SY5Y) days. All cultures were analysed in parallel and fibroblast cultures differed by no more than 2 passages. Prior to experimentation, cells were plated onto glass coverslips (for Ca^2+^ imaging and immunocytochemistry) or directly into tissue culture flasks (for western blotting). For SH-SY5Y cells, glass coverslips were coated with 20 μg/mL poly-l-lysine.

### 2.3 Ca^2+^ imaging

Ca^2+^ imaging was performed using the fluorescent Ca^2+^ indicator Fura-2 as described in [6] using HEPES-buffered saline (HBS) consisting of 10 mM HEPES, 2 mM MgSO_4_, 156 mM NaCl, 3 mM KCl, 2 mM CaCl_2_, 1.25 mM KH_2_PO_4_ and 10 mM glucose (pH 7.4). Cells were stimulated with thapsigargin (Merck), cADPR-AM, synthesised as described previously [23] and GPN (glycyl-l-phenylalanine 2-naphthylamide, SantaCruz Biotech). Where indicated, extracellular Ca^2+^ was replaced with 1 mM EGTA.

### 2.4 Western blotting

Western blotting was performed as described in [24]. Blots were incubated with either mouse anti-β-glucocerebrosidase (overnight at 4 °C, diluted 1:500, EMD Millipore), mouse anti-LAMP1 (1 h at RT, diluted 1:500, Santa Cruz Biotechnology) or rabbit-anti-LC3II (overnight at 4 °C, diluted 1:1000, Cell Signalling) primary antibodies. Blots were stripped and re-probed with a goat anti-actin (1 h at RT, diluted 1:500, Santa Cruz Biotechnology) primary antibody. Anti-mouse (Santa Cruz Biotechnology), anti-rabbit (Bio-Rad) or anti-goat (Santa Cruz Biotechnology) IgG conjugated to horse-radish peroxidase were used as the secondary antibodies (1 h at RT, 1:2000).

### 2.5 Other methods

β-Glucocerebrosidase and β-hexosaminidase enzyme activities were measured using 4-methylumbelliferyl-β-d-glucopyranoside and 4-methylumbelliferyl-N-acetyl-glucosaminide, respectively as described in [22]. Immunocytochemistry using primary antibodies raised to LAMP1 (mouse, 1 h at 37 °C; diluted 1:10, Developmental Studies Hybridoma Bank H4A3 clone supernatant) or LAMP2 (mouse, 1 h at 37 °C, diluted 1:100, Santa Cruz Biotechnology), Lysotracker™ Red staining and confocal microscopy were performed as described in [24], [25].

### 2.6 Data analysis

The magnitude of Ca^2+^ release was calculated by subtracting the basal Fura-2 fluorescence ratio prior to stimulation (60 s of data acquisition) from the peak response. The area under the curve was estimated by summating the increases in fluorescence ratio following stimulation over a given period. For thapsigargin, the periods were 750 s and 400 s for fibroblasts and SH-SY5Y cells, respectively. For GPN, the period was 400 s. These analyses were done at the individual cell level over the entire field of view (typically 15 cells). Data were derived from the number of passages stated in the figure legends, averaged over multiple fields of view (*n*, stated in the figure panels) and presented as mean ± standard error of the mean. Statistical analyses were performed using Minitab 17. Independent-samples *t*-tests were applied and in the case of multiple comparisons, ANOVA analysis followed by a post hoc Tukey test. *p* < 0.05 was considered statistically significant.

## 3 Results

### 3.1 ER Ca^2+^ release is disrupted in GD and PD fibroblasts

**Fig. 1:**
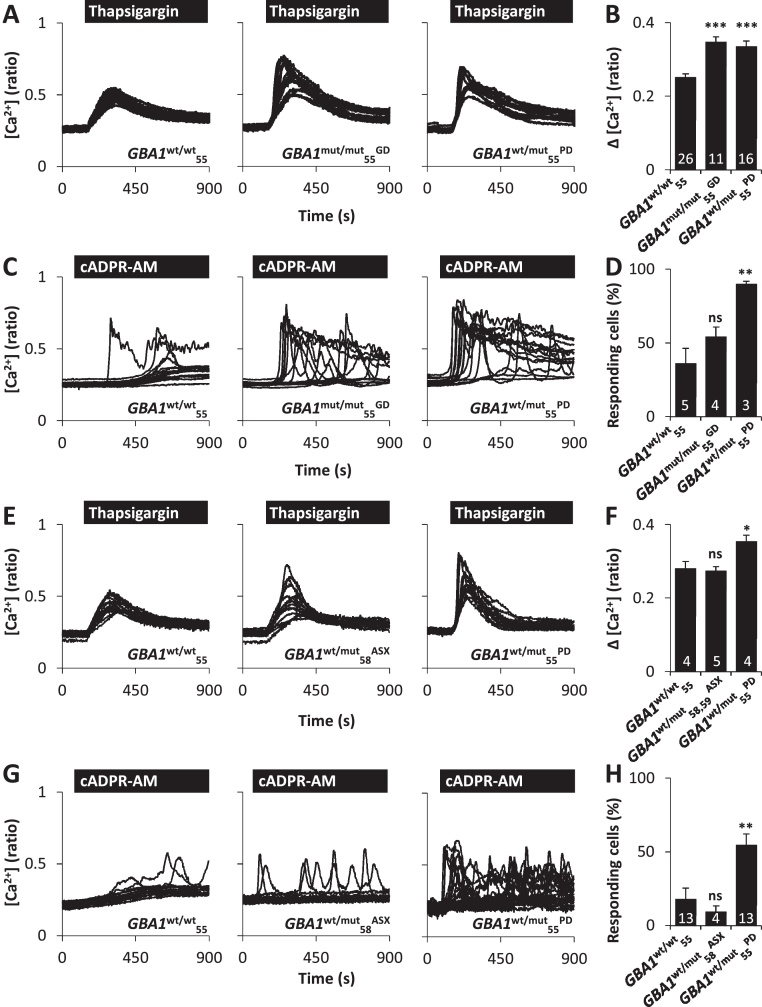
Pathogenic *GBA1* disrupts ER Ca^2+^ release. (A–D) ER Ca^2+^ release in *GBA1***^wt/wt^_55_**, *GBA1***^mut/mut^_55_^GD^** and *GBA1***^wt/mut^_55_^PD^** cells (young cohort). (A) Cytosolic Ca^2+^ recordings from individual fibroblasts challenged with thapsigargin (1 μM) from the indicated representative populations. Experiments were performed in the absence of extracellular Ca^2+^. (B) Summary data (mean ± SEM) quantifying the magnitude of thapsigargin-evoked Ca^2+^ signals in the indicated number of fields of view. Results are from 5 to 9 independent passages analysing 154–367 cells. (C) Cytosolic Ca^2+^ recordings from individual fibroblasts stimulated with cADPR-AM (25 μM). Experiments were performed in the presence of extracellular Ca^2+^. (D) Summary data quantifying the percentage of cells responsive to cADPR. Results are from 2 to 3 independent passages analysing 39–75 cells. (E) Similar to A except thapsigargin-evoked Ca^2+^ release was assessed in *GBA1***^wt/wt^_55_**, *GBA1***^wt/mut^_58_^ASX^** and *GBA1***^wt/mut^_55_^PD^** cells. (F) Summary data from 4 independent passages analysing 46–127 cells. (G) Similar to C except cADPR-evoked Ca^2+^ release was assessed in *GBA1***^wt/wt^_55_**, *GBA1***^wt/mut^_58_^ASX^** and *GBA1***^wt/mut^_55_^PD^** cells. (H) Summary data from 3 to 6 independent passages analysing 73–257 cells. *p < 0.05, **p < 0.01, ***p < 0.001, ns, not significant.

To examine whether GD and PD pathology is associated with impaired Ca^2+^ signalling, cytosolic Ca^2+^ levels were measured in age-segregated, passage-matched patient fibroblasts carrying the N370S mutation (see Section 2). In the first set of experiments, cultures from the younger cohort were used. We estimated ER Ca^2+^ content by challenging cells with the SERCA inhibitor thapsigargin (1 μM) in Ca^2+^-free medium. Thapsigargin-evoked Ca^2+^ release was significantly elevated in GD (*GBA1*^**mut/mut**^_**55**_^**GD**^) and PD (*GBA1*^**wt/mut**^_**55**_^**PD**^) cells when compared to cells from an age-matched (55 year old) healthy individual (*GBA1*^**wt/wt**^_**55**_) (Fig. 1A). These differences were quantified by measuring the magnitude of the response (Fig. 1B) or the area under the curve (Fig. S1A). To further examine ER Ca^2+^ release, fibroblasts were stimulated with a cell-permeable derivative of the intracellular Ca^2+^-mobilising messenger cyclic-ADP ribose (cADPR-AM) [23]. cADPR-AM (25 μM) evoked Ca^2+^ signals in a proportion of fibroblasts (Fig. 1C). The percentage of cells that responded to cADPR-AM was significantly increased in *GBA1*^**wt/mut**^_**55**_^**PD**^ fibroblasts compared to fibroblasts from an age-matched healthy individual (Fig. 1D). These data identify defects in ER Ca^2+^ release in both GD and *GBA1*-linked PD.

A significant number of individuals with heterozygous mutations in *GBA1* never develop neurological conditions [9]. ER Ca^2+^ release was therefore assessed in asymptomatic individuals with heterozygotic mutations in *GBA1*. Although thapsigargin-evoked Ca^2+^ release appeared more heterogeneous in *GBA1*^**wt/mut**^_**58**_^**ASX**^ when compared with control *GBA1*^**wt/wt**^_**55**_ fibroblasts (Fig. 1E), the mean amplitude of the Ca^2+^ elevations and the area under the curve did not differ between these cultures and those from an additional asymptomatic individual (*GBA1*^**wt/mut**^_**59**_^**ASX**^; Fig. 1F, Fig. S1A). Similarly, as shown in Fig. 1G-H, cADPR-AM-evoked Ca^2+^ release in *GBA1*^**wt/mut**^_**58**_^**ASX**^ fibroblasts was not significantly different to control *GBA1*^**wt/wt**^_**55**_ fibroblasts. These data suggest that disrupted Ca^2+^ homeostasis correlates with PD in the same *GBA1* genetic background.

### 3.2 ER Ca^2+^ defects are age-dependent

**Fig. 2:**
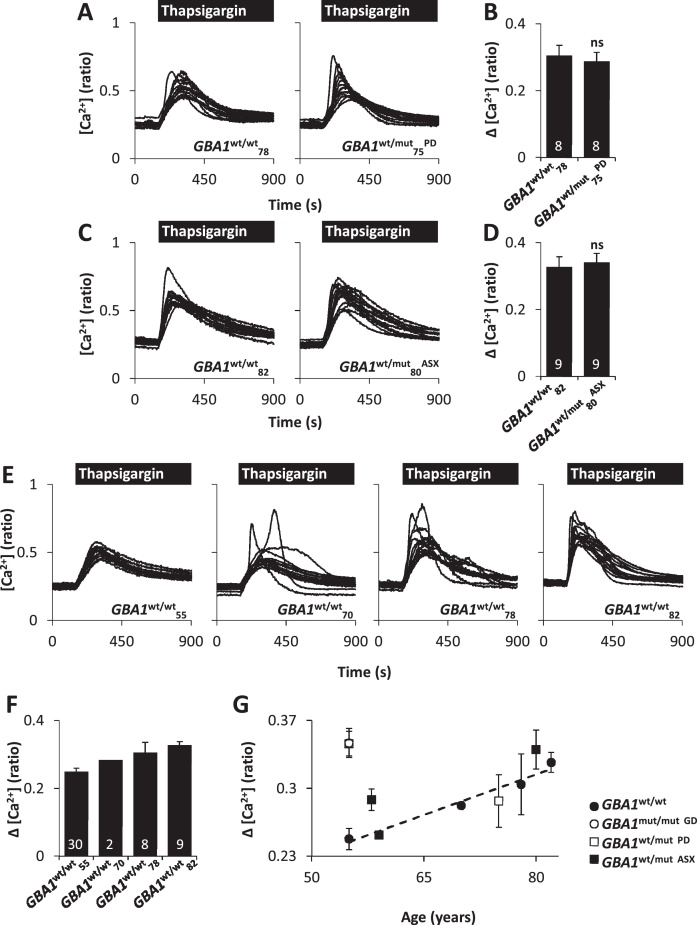
ER Ca^2+^ defects are age-dependent. (A) Cytosolic Ca^2+^ recordings from individual fibroblasts challenged with thapsigargin (1 μM) from representative populations of *GBA1***^wt/wt^_78_** and *GBA1***^wt/mut^_75_^PD^** cells (aged cohort). (B) Summary data 3 independent passages analysing 112–117 cells. (C) Similar to A except, ER Ca^2+^ release was assessed in *GBA1***^wt/wt^_82_** and *GBA1***^wt/mut^_80_^ASX^** cells. (D) Summary data from 3 independent passages analysing 131–134 cells. (E) ER Ca^2+^ release from *GBA1***^wt/wt^** fibroblasts with increasing age. (F) Summary data from 1 to 14 independent passages analysing 30–483 cells. (G) Magnitude of ER Ca^2+^ release versus age for both the young and aged cohort. All experiments were performed in the absence of extracellular Ca^2+^.

ER Ca^2+^ release in PD was further examined using fibroblasts from the aged cohort. Unlike the younger *GBA1*^**wt/mut**^_**55**_^**PD**^ fibroblasts, thapsigargin-evoked Ca^2+^ release in *GBA1*^**wt/mut**^_**75**_^**PD**^ fibroblasts was similar to fibroblasts from the age-matched healthy control (*GBA1*^**wt/wt**^_**78**_) (Fig. 2A and B, Fig. S1A). However, we noted that thapsigargin-evoked Ca^2+^ release in fibroblasts from both *GBA1*^**wt/wt**^_**78**_ and *GBA1*^**wt/mut**^_**75**_^**PD**^ was kinetically irregular and larger than Ca^2+^ release evoked in fibroblasts from younger control subjects (compare with Fig. 1A). To investigate the effect of age on ER Ca^2+^ release, we examined the effects of thapsigargin in fibroblasts from healthy individuals of increasing age. As shown in Fig. 2E and F, thapsigargin-evoked Ca^2+^ release increased in an age-dependent manner in fibroblasts from control (*GBA1*^**wt/wt**^) individuals. Thapsigargin responses in the oldest fibroblasts examined (*GBA1*^**wt/wt**^_**82**_), closely resembled those from the younger *GBA1*^**wt/mut**^_**55**_^**PD**^ fibroblasts (Fig. 2G). Such findings are consistent with the idea that some features of PD simulate an accelerated form of ageing [26].

### 3.3 ER Ca^2+^ defects are independent of β-glucocerebrosidase activity loss

**Fig. 3:**
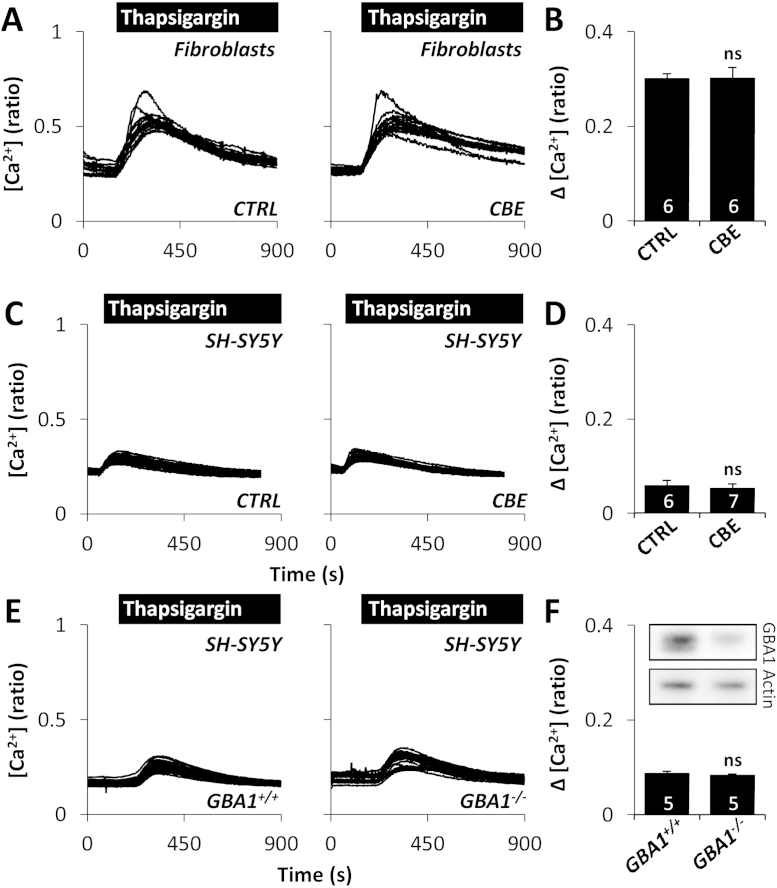
Inhibition of β-glucocerebrosidase does not affect ER Ca^2+^ release. (A) Cytosolic Ca^2+^ recordings from individual control *GBA1***^wt/wt^** fibroblasts challenged with thapsigargin (1 μM) from a representative population treated with 10 μM CBE for 8 days. (B) Summary data from 2 independent treatments analysing 87–90 cells. (C–F) Cytosolic Ca^2+^ recordings from individual SH-SY5Y cells challenged with thapsigargin (1 μM) from a representative population treated with 10 μM CBE for 10–11 days (C) or stably expressing either scrambled shRNA (*GBA1*^+/+^) or shRNA targeting *GBA1* (*GBA1*^−/−^) (E). Summary data from 3 independent treatments analysing 117–204 cells (D) and 3 independent passages analysing 150–143 cells (F). All experiments were performed in the absence of extracellular Ca^2+^. ns, not significant. Inset (F) is a Western blot using antibodies to β-glucocerebrosidase (top) or actin (bottom) and homogenates (14 μg) from SH-SY5Y cells treated with the indicated shRNA.

Whether pathogenic effects of *GBA1* are due to loss of enzymatic function or gain of toxic function is debated [27]. To probe the mechanism of how mutant *GBA1* disrupts ER Ca^2+^ release, the effects of thapsigargin were examined in fibroblasts from healthy controls by reducing the activity of β-glucocerebrosidase using pharmacological and molecular means. Fibroblasts were chronically treated with conduritol B epoxide (CBE, 10 μM), an inhibitor of β-glucocerebrosidase, which reduced β-glucocerebrosidase activity to 6 ± 0.03%. Thapsigargin-induced Ca^2+^ release after exposure to CBE was unchanged (Fig. 3A and B, Fig. S1B). To extend these studies to a more neuronal context, we examined the effect of CBE on dopaminergic SH-SY5Y cells. As in fibroblasts, thapsigargin-evoked Ca^2+^ release was not different following CBE treatment (Fig. 3C and D, Fig. S1B) despite substantial reduction in β-glucocerebrosidase enzyme activity to 8 ± 0.4%. To probe further the role of β-glucocerebrosidase, we examined the effect of thapsigargin upon stable knockdown of *GBA1* [22]. Reducing the levels of β-glucocerebrosidase did not affect thapsigargin-evoked Ca^2+^ release (Fig. 3E and F, Fig. S1B). Taken together, these data show that reducing β-glucocerebrosidase enzyme activity, under our experimental conditions, appears not to induce ER Ca^2+^ dysfunction.

### 3.4 Lysosomal morphology and Ca^2+^ content is disrupted in GD and PD fibroblasts

**Fig. 4:**
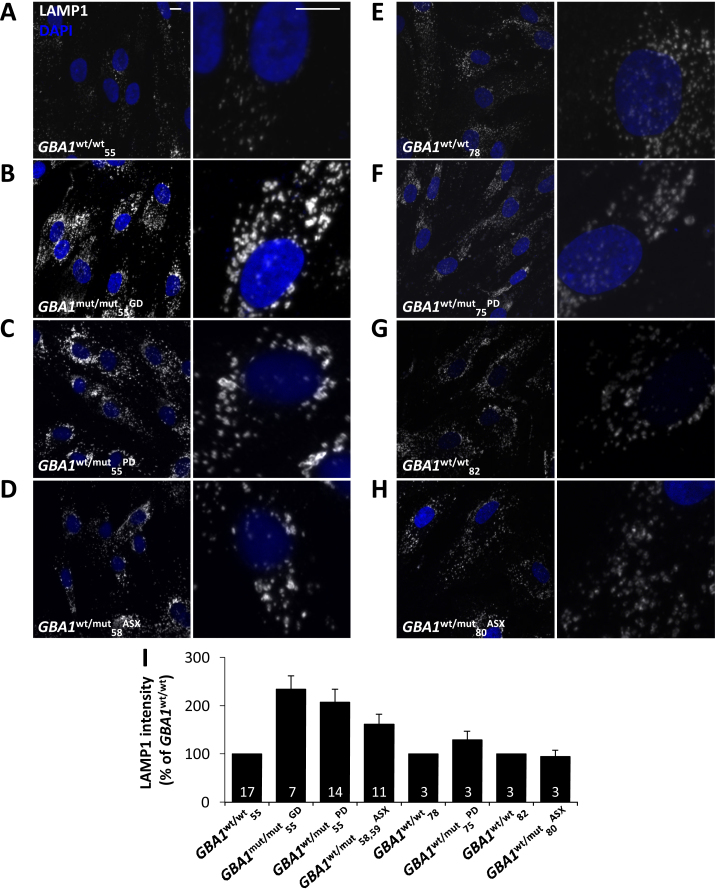
Pathogenic *GBA1* disrupts lysosomal morphology. (A–H) Representative confocal fluorescence images of LAMP1 staining (white) in the indicated fibroblasts from the young (A–D) and aged (E–H) cohort. Nuclei were stained with DAPI (blue). Zoomed images are displayed in the right panels. Scale bars, 10 μm. (I) Summary data quantifying LAMP1 intensity as a percentage of the indicated age-matched control (82–654 cells). (For interpretation of the references to color in this figure legend, the reader is referred to the web version of this article.)

Lysosomes are increasingly implicated in PD pathogenesis [10], [11]. We recently identified lysosome morphology defects in *LRRK2*-PD fibroblasts which we correlated with lysosomal Ca^2+^ defects [24]. We therefore probed potential physical and functional lysosome alterations in *GBA1*-PD fibroblasts. Using an antibody raised to the late endosome/lysosome marker LAMP1, lysosome morphology was compared in the fibroblasts from the young and aged cohorts (Fig. 4). Lysosome morphology was altered in the *GBA1*^**mut/mut**^_**55**_^**GD**^ fibroblasts (Fig. 4B) compared to age-matched control fibroblasts (*GBA1*^**wt/wt**^_**55**_; Fig. 4A). Lysosome morphology was also altered in *GBA1*^**wt/mut**^_**55**_^**PD**^ fibroblasts (Fig. 4C). In both cases, lysosomes appeared enlarged and clustered. Similar morphological alterations were apparent in the GD and PD cells using an antibody raised to LAMP2 (Fig. S2A–C and F) and in live cells labelled with the acidotrope, Lysotracker (Fig. S2D–F). There was little change in LAMP1 protein levels quantified by Western blotting in either GD or PD fibroblasts consistent with our previous analysis [21], although levels of the autophagic marker LC3II were increased (Fig. S2G).

Morphological alterations to the lysosomal system were also found in asymptomatic *GBA1* carriers (*GBA1*^**wt/mut**^_**58**_^**ASX**^ and *GBA1*^**wt/mut**^_**59**_^**ASX**^) but to a lesser extent than in GD and PD fibroblasts (Fig. 4D and data not shown). Importantly, lysosome morphology did not differ in healthy, PD and asymptomatic carriers from the aged cohort (Fig. 4E–H). These data are summarised in Fig. 4I. Thus, similar to ER Ca^2+^ defects, lysosome morphology defects are age-dependent.

**Fig. 5:**
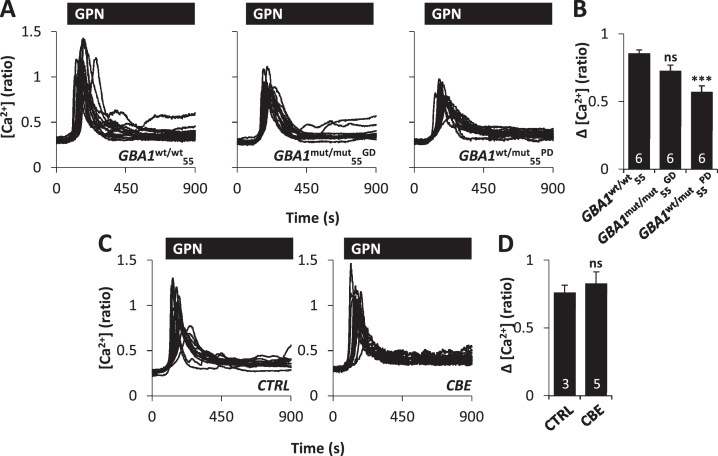
Pathogenic *GBA1* disrupts lysosomal Ca^2+^ content. (A–D) Cytosolic Ca^2+^ measurements from individual fibroblasts stimulated with GPN (200 μM). Experiments were performed in the presence of extracellular Ca^2+^ and following a 12.5 min pre-treatment with 100 μM 2APB prior to recording. (A) Recordings from a representative population of *GBA1***^wt/wt^_55_**, *GBA1***^mut/mut^_55_^GD^** and *GBA1***^wt/mut^_55_^PD^** cells. (B) Summary data from 2 independent passages analysing 72–88 cells. (C) Recordings from a representative population of control *GBA1***^wt/wt^** fibroblasts treated with 10 μM CBE for 7–9 days. (D) Summary data from 2 independent treatments analysing 43–72 cells. ***p < 0.001. ns, not significant.

To estimate lysosomal Ca^2+^ content, we challenged cells with the lysosomotropic agent GPN (200 μM) which induces leak of low molecular weight solutes (<10 kDa) in fibroblasts upon hydrolysis by the lysosomal protease, cathepsin C [28]. GPN stimulated complex cytosolic Ca^2+^ increases, as reported previously [6], and no differences were observed across the *GBA1*^**wt/wt**^_**55**_, *GBA1*^**mut/mut**^_**55**_^**GD**^ and *GBA1*^**wt/mut**^_**55**_^**PD**^ fibroblast cultures (Fig. S3A and B). Potential differences in lysosomal Ca^2+^ content may have been masked due to recruitment of ER-localised receptors upon lysosomal destabilisation [6]. Indeed, in human fibroblasts we have previously shown that lysosomal Ca^2+^ release triggers Ca^2+^ responses through IP_3_, but not ryanodine, receptors [6]. We therefore isolated lysosomal Ca^2+^ release by blocking IP_3_ receptors with 2-APB prior to GPN challenge. Under these conditions, GPN-evoked Ca^2+^ release was largely monotonic and reduced in *GBA1*^**wt/mut**^_**55**_^**PD**^ fibroblasts relative to controls (Fig. 5A and B, Fig. S1C). This reduction was not due to differences in cathepsin C activity/lysosomal permeabilisation because the rate of fluorescence loss in cells loaded with Lysotracker in response to GPN, was similar between fibroblasts cultures (Fig. S3C and D). Activity of β-hexosaminidase was also unchanged in GD and PD cells (106 ± 10% and 103 ± 3% of control, respectively). Similar to our ER Ca^2+^ estimates, reducing the activity of β-glucocerebrosidase with CBE had little effect on GPN-evoked Ca^2+^ release in fibroblasts from healthy controls (Fig. 5C and D). We therefore identify Ca^2+^ defects at the lysosomal level in PD that are likely independent of β-glucocerebrosidase activity loss.

## 4 Discussion

Ca^2+^ stores represent a major source of Ca^2+^ signals but their role in PD is largely unknown. In the present study, we identify age-dependent changes in ER Ca^2+^ release in both type I GD and *GBA1*-linked PD fibroblasts. Additionally, we report disturbances in lysosomal morphology and lysosomal Ca^2+^ content in these cells.

Patient fibroblasts represent a robust, tractable system for disease study. They harbour cumulative damage for a given subject, perhaps particularly relevant to late onset neurodegenerative disease. Nevertheless, a limitation of fibroblasts is their non-neuronal nature. Our data demonstrating exaggerated ER Ca^2+^ release in *GBA1*-PD fibroblasts however is consistent with a recent report using induced pluripotent stem cell-derived dopaminergic neurons which showed enhanced Ca^2+^ release to the ryanodine receptor agonist, caffeine [29]. In that study, lines were derived from patients with an L444P mutation in *GBA1* and asymptomatic carriers were unavailable. Defects reported here were not manifest in asymptomatic carriers and presented only in the younger patients. We interpret the defect as being “non-additive” with ageing which we report is also associated with similar perturbations in ER Ca^2+^ signalling. Notably, strategies that increase ER Ca^2+^ content improve mutant β-glucocerebrosidase folding [30]. Enhanced ER Ca^2+^ content, although beneficial with respect to protein folding, may render cells more sensitive to apoptotic stimuli and thus link Ca^2+^ disturbances to cell death.

Reduced lysosomal Ca^2+^ content in *GBA1*-PD fibroblasts is similar to that reported in Niemann–Pick type C1 diseased fibroblasts [16] and Presenilin-1 knockout mouse embryonic fibroblasts [31], [32]. Functionally, reduced lysosomal Ca^2+^ content might affect Ca^2+^-dependent membrane trafficking events within the endo-lysosomal system [33], thereby accounting for altered lysosome morphology. Similar lysosome morphology alterations have been reported in fibroblasts from patients with mutations in ATP13A2 (*PARK9*) [34], a lysosomal ATPase and LRRK2 (*PARK8*) for which evidence of an endolysosomal locus of action continues to accrue [24], [35], [36], [37].

How mutant *GBA1* disposes to PD is unclear. Both loss- and gain-of function models have been proposed [27]. For-example, knock-down of β-glucocerebrosidase in mouse models is associated with increases in the substrate glucocerebroside which stabilises α-synuclein, a component of Lewy bodies characteristic of the disease [38]. Concomitantly, α-synuclein also reduces trafficking of β-glucocerebrosidase to the lysosome pointing to a positive feedback loop triggered by a reduction in β-glucocerebrosidase activity that might precipitate disease [38]. However, increases in α-synuclein levels do not always correlate with β-glucocerebrosidase activity [27]. Notably, the E326K mutation in β-glucocerebrosidase, which is linked to early onset PD, has a more modest effect on β-glucocerebrosidase activity than other mutations and does not cause Gaucher disease [39]. Furthermore, we have shown that substrate does not accumulate in the brains of *GBA1* carrier-PD patients [40] despite demonstrable reduction in β-glucocerebrosidase activity [41]. That many mutant forms of β-glucocerebrosidase accumulate in the ER supports the alternative gain-of function mechanism for toxicity [27]. Our findings reported here, showing that neither ER nor lysosomal defects were recapitulated upon inhibiting/depleting β-glucocerebrosidase, support such a gain-of-function mechanism for pathogenic *GBA1*. However, we cannot rule out that residual β-glucocerebrosidase activity (albeit modest) is sufficient to maintain homeostasis.

PD has a complex aetiopathogenesis, which likely results from interplay between genetic and environmental cues. Although it is established that mutations in *GBA1* substantially increase risk of developing PD not all carriers succumb. These data strongly suggest that pathology is not a sole consequence of the mutant *GBA1* allele. We show here that ER Ca^2+^ and lysosomal morphology defects identified in PD cells are not present in asymptomatic *GBA1* carriers. Thus, defects correlate with pathology despite similar *GBA1* status. It remains to be established whether these phenotypes are contributing causal factors for the disease or a consequence. Nevertheless, disruptions in fibroblast Ca^2+^ store homeostasis and lysosomal morphology described here might serve as biomarkers for *GBA1*-linked PD given incomplete penetrance in *GBA1* carriers. Further work, however, is required using additional patient cell lines to validate our findings.

In summary, we identify age-dependent disturbances in both ER and lysosomal Ca^2+^ stores of potential relevance to the pathology of PD and GD.

## Author contributions

BSK performed the Ca^2+^ and Lysotracker imaging, immunocytochemistry and Western blotting. MEG and BSK performed enzyme activity measurements, JM and MJWC provided the SH-SY5Y cells. MSB and AM obtained the fibroblasts. DB and GCC synthesised the cADPR-AM. MRD, AHS and SP conceived the study. BSK and SP wrote the paper with input from all authors.

## Funding

This work was supported by an IMPACT studentship from UCL (to BSK), Parkinson's UK grants K-1107, K-1412 and H-1202 (to SP, AHS and MRD), Wellcome Trust/MRC Joint Call in Neurodegeneration award (WT089698 to AHS and MRD), an MRC CoEN award (to AHS). AHS is a NIHR Senior Investigator and is supported by the NIHR UCLH BRC.
