# Supplementary material for: Endoplasmic reticulum and lysosomal Ca2+ stores are remodelled in GBA1-linked Parkinson disease patient fibroblasts
Source: Cell Calcium. 2016 Jan;59(1):12–20. doi: 10.1016/j.ceca.2015.11.002 (PMC4751977; doi:10.1016/j.ceca.2015.11.002)
Supplement: Supplementary file 1 [file mmc1.pdf]

Supplementary Table S1. Details of human fibroblasts used in this study.

Category	Identifier	Sex	Age	Genotype	Clinical features
Young	$\mathit{GBA1}^{wt/wt}_{55}$	Female	55	WT/WT	Apparently healthy
	$\mathrm{GBA1}^{mut/mut\ \ GD}_{55}$	Male	55	N370S/ 1263del55	Type I Gaucher Disease
	$\mathit{GBA1}^{wt/mut\ \ PD}_{55}$	Female	55	WT/N370S	Parkinson Disease
	$\mathit{GBA1}^{wt/mut\ \ ASX}_{58}$	Male	58	WT/N370S	Non-manifesting carrier
	$\mathit{GBA1}^{wt/mut\ \ ASX}_{59}$	Female	59	WT/N370S	Non-manifesting carrier
Aged	$\mathit{GBA1}^{wt/wt}_{70}$	Female	70	WT/WT	Apparently healthy
	$\mathit{GBA1}^{wt/wt}_{78}$	Male	78	WT/WT	Apparently healthy
	$\mathit{GBA1}^{wt/mut\ \ PD}_{75}$	Female	75	WT/N370S	Parkinson Disease
	$\mathit{GBA1}^{wt/wt}_{82}$	Female	82	WT/WT	Apparently healthy
	$\mathit{GBA1}^{wt/mut\ \ ASX}_{80}$	Female	80	WT/N370S	Non-manifesting carrier

Fig. S1. Pathogenic *GBA1* disrupts ER and lysosomal Ca^{2+} content. Summary data quantifying area under the curve measurements in cells stimulated with thapsigargin (**A-B**) or GPN (**C**). **A**, Thapsigargin responses in fibroblasts upon *GBA1* mutation. Data are expressed as a percentage of the indicated age-matched control and correspond to experiments described in Figures 1-2. **B**, Thapsigargin responses upon β-glucocerebrosidase inhibition. Data from fibroblasts (left) or SHSY5Y cells (middle, right) are expressed as a percentage of control cells not treated with CBE or cells expressing scrambled shRNA (*GBA1*$^{+/+}$) and correspond to experiments described in Fig. 3. **C**, GPN responses in fibroblasts upon *GBA1* mutation or CBE treatment. Data are expressed as in B and correspond to experiments described in Fig. 5.

Fig. S2. Pathogenic *GBA1* disrupts lysosomal morphology. A-C, Representative confocal fluorescence images of LAMP2 staining (white) in *GBA1*$^{\mathbf{wt/wt}}_{55}$, *GBA1*$^{\mathbf{mut/mut}}_{55}{}^{\mathbf{GD}}$ and *GBA1*$^{\mathbf{wt/mut}}_{55}{}^{\mathbf{PD}}$ cells. Nuclei were stained with DAPI (blue). Zoomed images are displayed in the right panels. Scale bars, 10 µm. **D-E**, Representative confocal fluorescence images of live *GBA1*$^{\mathbf{wt/wt}}_{55}$ and *GBA1*$^{\mathbf{wt/mut}}_{55}{}^{\mathbf{PD}}$ cells labelled with Lysotracker red (100 nM, 15 min.). **F**, Summary data quantifying LAMP2 and Lysotracker intensity as a percentage of the control (48-130 cells). **G**, Western blot analysis using antibodies to LAMP1 (left) or LC3 (right) and homogenates prepared from *GBA1*$^{\mathbf{wt/wt}}_{55}$, *GBA1*$^{\mathbf{mut/mut}}_{55}{}^{\mathbf{GD}}$ and *GBA1*$^{\mathbf{wt/mut}}_{55}{}^{\mathbf{PD}}$ cells (21 µg). Blots were stripped and re-probed using an antibody to actin (bottom panels). Migration of molecular mass markers (in kDa) is shown on the right of the panels. Migration of LC3II is highlighted. Blots are representative of those using 3 independent homogenate preparations.

Fig. S3. Effect of GPN on cytosolic Ca^{2+} and lysosome integrity. A, Cytosolic Ca^{2+} release from individual fibroblasts stimulated with GPN (200 µM) from a representative population of *GBA1*$^{\mathbf{wt/wt}}_{55}$, *GBA1*$^{\mathbf{mut/mut}}_{55}{}^{\mathbf{GD}}$ and *GBA1*$^{\mathbf{wt/mut}}_{55}{}^{\mathbf{PD}}$ cells. **B,** Summary data quantifying percentage of cells that oscillated in response to GPN. Data are from 2 independent passages analysing 118-120 cells. **C**, Lysotracker red responses of fibroblasts (representative population average) stimulated with GPN (200 µM). **D**, Summary data quantifying the time taken to reach a half-maximal loss of Lysotracker red fluorescence (25-34 cells).

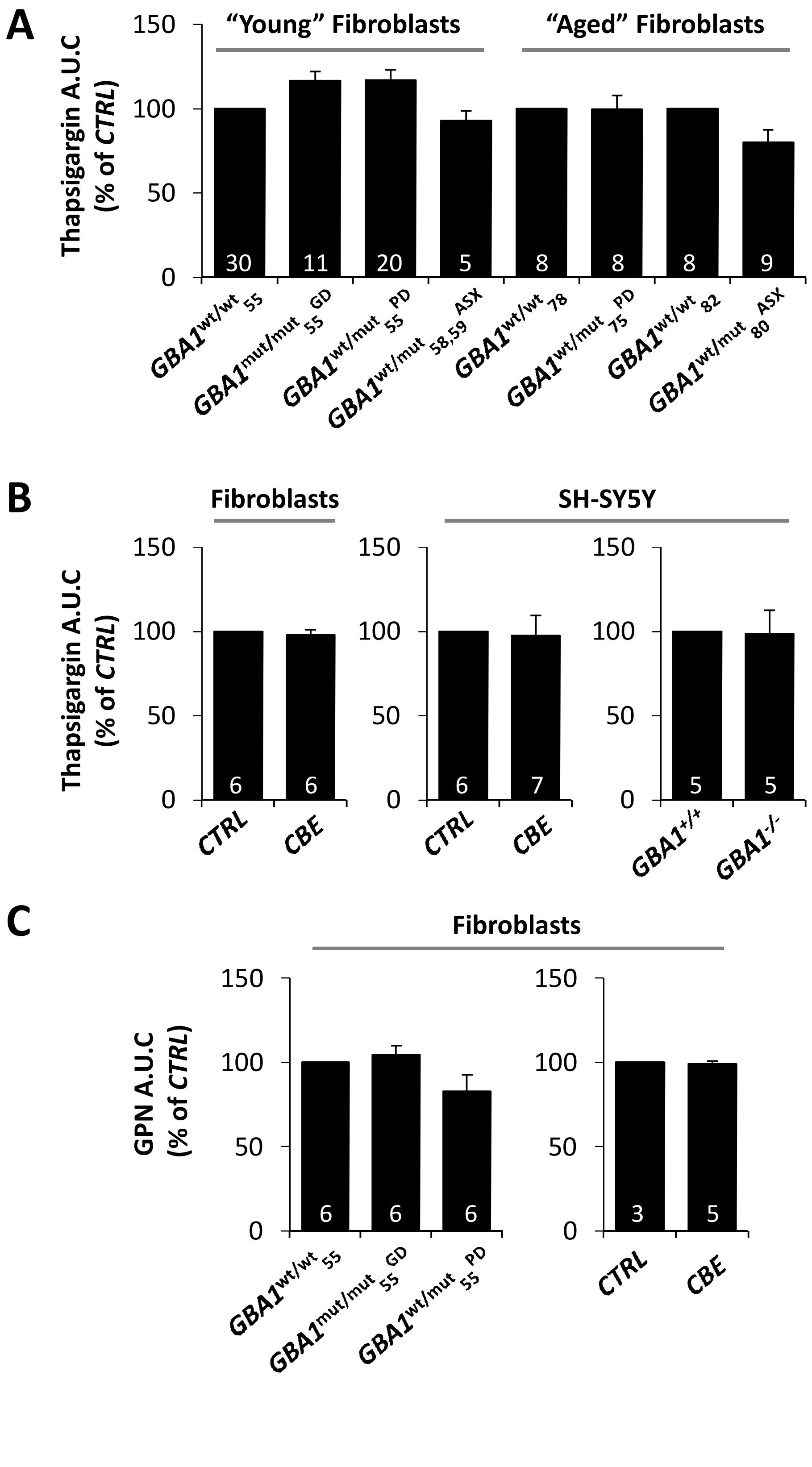
A
"Young" Fibroblasts
"Aged" Fibroblasts
Thapsigargin A.U.C
(% of CTRL)
150
100
50
0
30
11
20
5
8
8
8
9
GBA1wt/wt 55
GBA1mut/mut GD 55
GBA1wt/mut PD 55
GBA1wt/mut ASX 58,59
GBA1wt/wt 78
GBA1wt/mut PD 75
GBA1wt/wt 82
GBA1wt/mut ASX 80
B
Fibroblasts
SH-SY5Y
6
6
6
7
5
5
CTRL
CBE
GBA1+/+
GBA1-/-
C
Fibroblasts
GPN A.U.C
(% of CTRL)
3
5

Fig. S1.

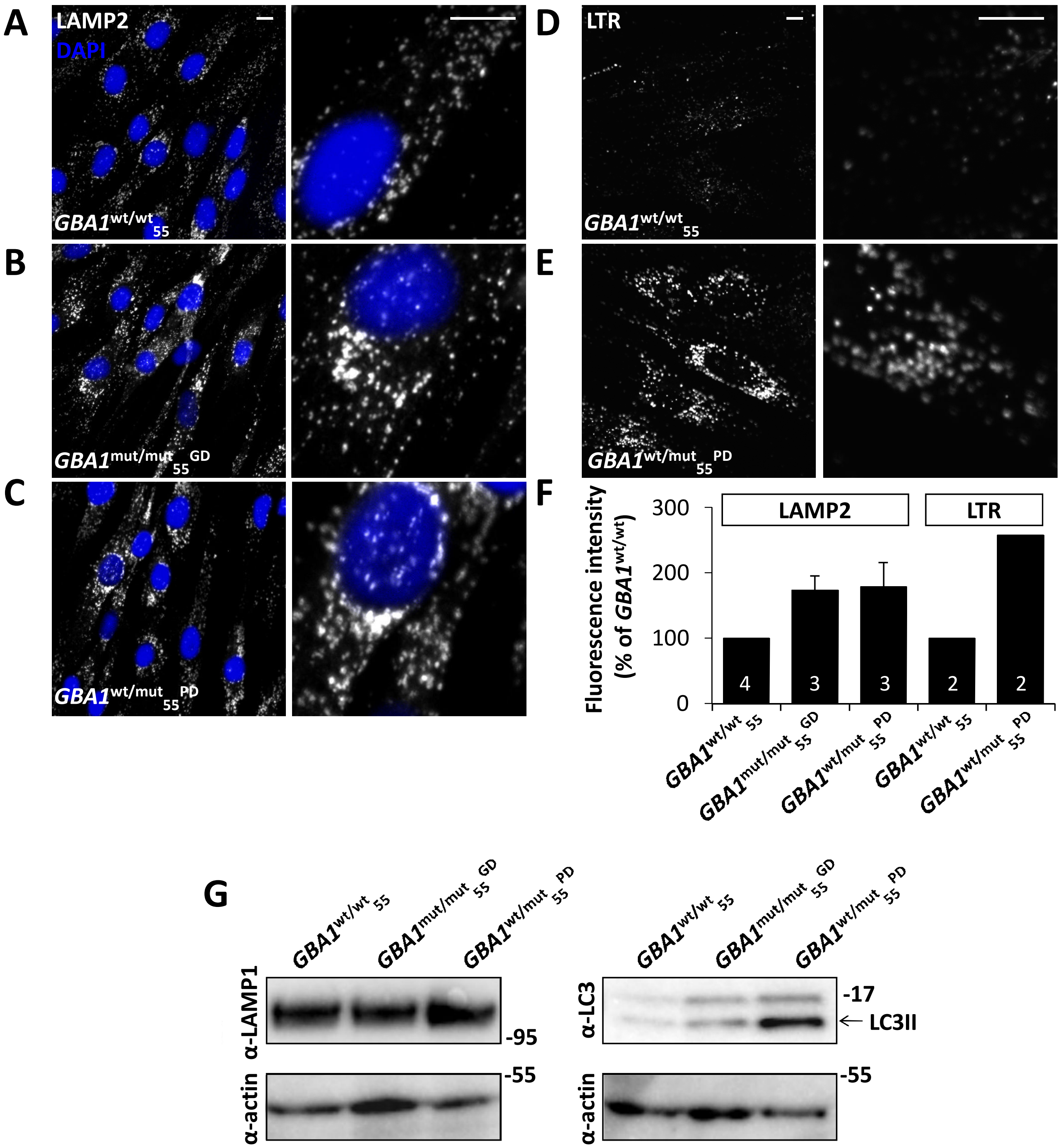
A
LAMP2
DAPI
GBA1wt/wt 55
B
GBA1mut/mut 55 GD
C
GBA1wt/mut 55 PD
D
LTR
GBA1wt/wt 55
E
GBA1wt/mut 55 PD
F
Fluorescence intensity (% of GBA1wt/wt)
LAMP2
LTR
300
200
100
0
4
3
3
2
2
GBA1wt/wt 55
GBA1mut/mut 55 GD
GBA1wt/mut 55 PD
GBA1wt/wt 55
GBA1wt/mut 55 PD
G
GBA1wt/wt 55
GBA1mut/mut 55 GD
GBA1wt/mut 55 PD
α-LAMP1
-95
α-actin
-55
α-LC3
-17
LC3II
α-actin
-55

Fig. S2.

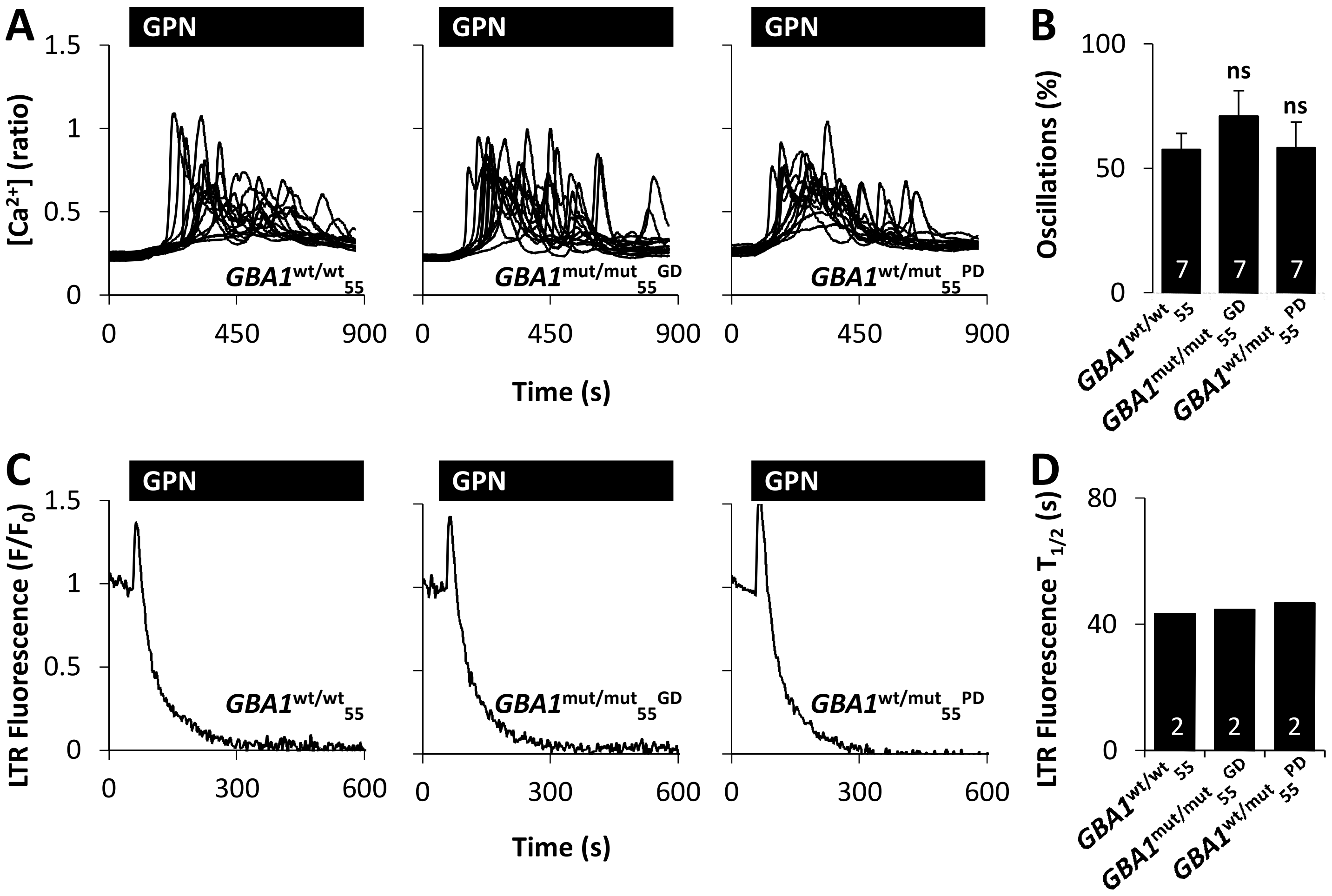
A
B
C
D
GPN
[Ca2+] (ratio)
1.5
1
0.5
0
GBA1wt/wt55
GBA1mut/mut55GD
GBA1wt/mut55PD
0
450
900
Time (s)
Oscillations (%)
100
50
ns
7
LTR Fluorescence (F/F0)
300
600
LTR Fluorescence T1/2 (s)
80
40
2

Fig. S3.
